# Single locus affects embryonic segment polarity and multiple aspects of an adult evolutionary novelty

**DOI:** 10.1186/1741-7007-8-111

**Published:** 2010-08-26

**Authors:** Suzanne V Saenko, Paul M Brakefield, Patrícia Beldade

**Affiliations:** 1 Institute of Biology, Leiden University, Sylviusweg 72, 2333 BE Leiden, The Netherlands; 2 Instituto Gulbenkian de Ciência, Rua da Quinta Grande 6, P-2780-156 Oeiras, Portugal

## Abstract

**Background:**

The characterization of the molecular changes that underlie the origin and diversification of morphological novelties is a key challenge in evolutionary developmental biology. The evolution of such traits is thought to rely largely on co-option of a toolkit of conserved developmental genes that typically perform multiple functions. Mutations that affect both a universal developmental process and the formation of a novelty might shed light onto the genetics of traits not represented in model systems. Here we describe three pleiotropic mutations with large effects on a novel trait, butterfly eyespots, and on a conserved stage of embryogenesis, segment polarity.

**Results:**

We show that three mutations affecting eyespot size and/or colour composition in *Bicyclus anynana *butterflies occurred in the same locus, and that two of them are embryonic recessive lethal. Using surgical manipulations and analysis of gene expression patterns in developing wings, we demonstrate that the effects on eyespot morphology are due to changes in the epidermal response component of eyespot induction. Our analysis of morphology and of gene expression in mutant embryos shows that they have a typical segment polarity phenotype, consistent with the mutant locus encoding a negative regulator of Wingless signalling.

**Conclusions:**

This study characterizes the segregation and developmental effects of alleles at a single locus that controls the morphology of a lineage-specific trait (butterfly eyespots) and a conserved process (embryonic segment polarity and, specifically, the regulation of Wingless signalling). Because no gene with such function was found in the orthologous, highly syntenic genomic regions of two other lepidopterans, we hypothesize that our locus is a yet undescribed, possibly lineage-specific, negative regulator of the conserved Wnt/Wg pathway. Moreover, the fact that this locus interferes with multiple aspects of eyespot morphology and maps to a genomic region containing key wing pattern loci in different other butterfly species suggests it might correspond to a 'hotspot' locus in the diversification of this novel trait.

## Background

The origin and diversification of novel morphological traits, such as angiosperm flowers, bird feathers, insect wings, or beetle horns, have always fascinated biologists and laymen alike and are currently a key theme in evolutionary developmental biology [1,2]. Novelties arise during the evolution of a lineage and perform new functions within its ecology [3]. While the ecological and evolutionary factors that promote the diversification of such traits have been studied for some time (e.g., [4,5]), it is only more recently that the genetic and developmental mechanisms underlying their formation have become the focus of attention. Morphological novelties seem to arise largely through redeployment, or co-option, of conserved developmental toolkit genes (e.g., [6-9]), though recent studies suggest that taxonomically restricted genes might also be important [10].

Lepidoptera (the insect order of butterflies and moths) provide several examples of lineage-restricted traits that presumably evolved by co-option of genes or gene networks shared across insects. Signalling pathways and enzymes involved in the development of insect wings, sensory bristles or visual pigments have been implicated in the development [11,12] and coloration [13,14] of the scales that cover lepidopteran wings, and in the formation of particular pattern elements, the eyespots (reviewed in [15]). Eyespots have emerged as a promising model to investigate how novel characters arise [16-18], and which genes and components of pattern induction are modified in relation to phenotypic variation [19-22]. Pattern elements made of concentric rings of different colours are present on the wings of many butterflies and moths and show extreme intra- and interspecific variation in colour, size, shape and number [23,24]. Their adaptive role in predator avoidance [25-27] and mate choice [28,29] has been demonstrated in different species, and the mechanistic basis of eyespot diversity is now the focus of active research in evolutionary developmental biology [15,30].

Classical surgical manipulations of developing wings, such as tissue transplantation and damage, showed that eyespot centres, called foci, act as organizers of pattern formation [31,32]. In pupal wings, the focal cells presumably produce one or more diffusible morphogens, and the neighbouring epidermal cells respond to these signals in a concentration-dependent manner and become fated to synthesize particular pigments [23,33]. A number of conserved genes and signalling pathways with known functions in insect wing development have been implicated in the determination of eyespot foci [16,34] and colour rings [17,35]. Little, however, is known about which particular genes contribute to variation in eyespot morphology [19] and how these affect the signal/response components of eyespot induction.

Captive populations of the lab model *Bicyclus anynana *harbour different types of genetic variation affecting eyespot patterns [13], including spontaneous mutations of large phenotypic effect [36], some of which have been assigned to linkage groups [37]. Analysis of such mutations offers an opportunity to characterize the genetic and developmental bases of a novel trait not represented in classical model organisms (e.g., [17,18,38-41]). Of special interest are pleiotropic mutations that affect not only eyespots but also some other, conserved developmental processes. For example, *veinless *and *Cyclops *affect eyespot number and wing vein development, and *Goldeneye *disturbs eyespot colour composition and embryonic development [17,18]. Here, we characterize the effects of different alleles of a single locus on various aspects of eyespot morphology and on embryonic segment polarity. Our analysis suggests that this locus is a potentially novel negative regulator of the Wnt/Wg signalling pathway and a candidate 'hotspot' for wing pattern diversification.

## Results and Discussion

### Eyespot mutations are embryonic recessive lethal alleles at a single locus

**Figure 1:**
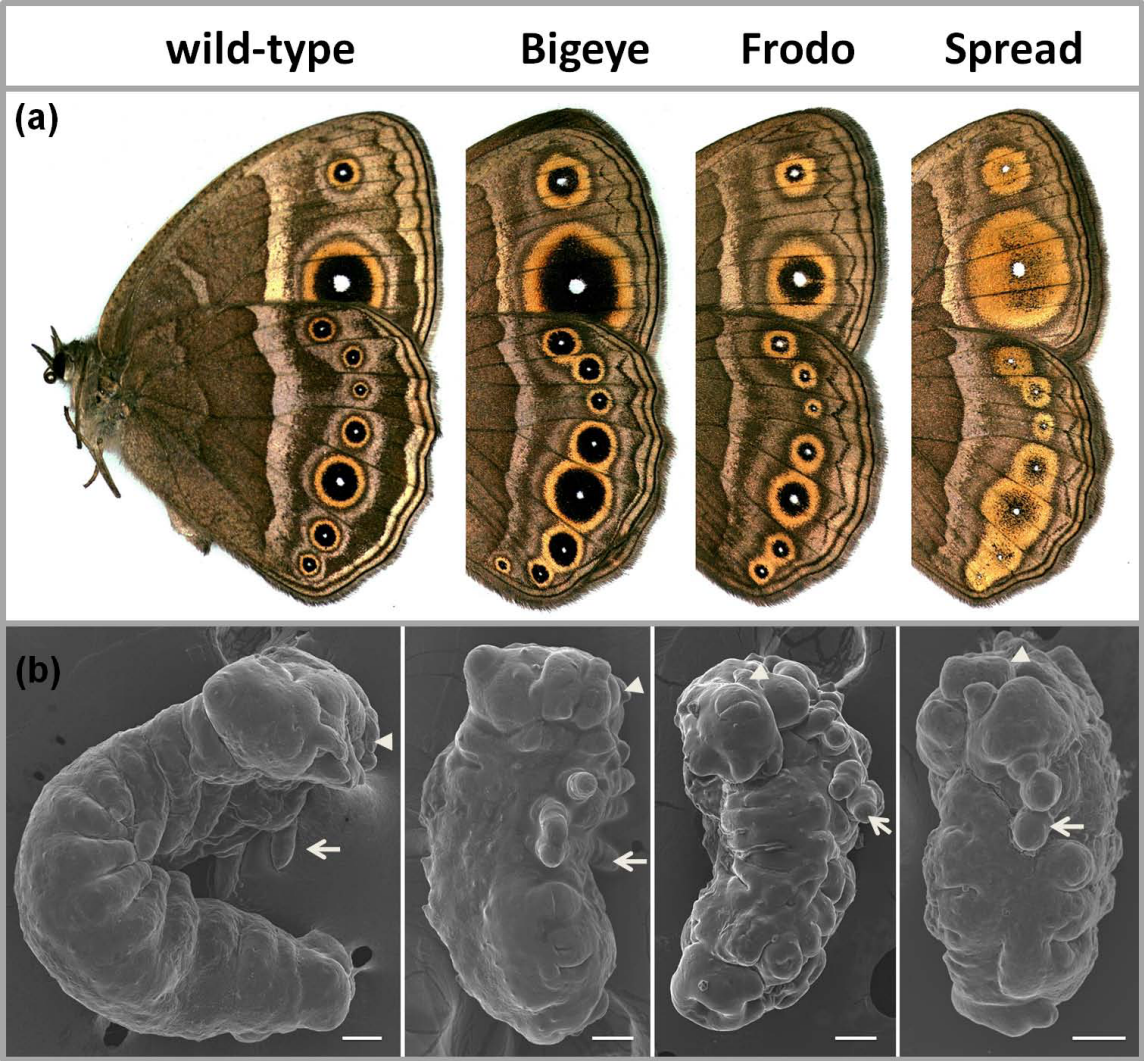
**Mutants with altered eyespots and disturbed embryogenesis**. (a) Representative images of the ventral surface of wings of WT and mutant *B. anynana *females. (b) Representative scanning electron microscopy images of WT and mutant embryos at ~60% DT (lateral view, anterior is up and dorsal is to the left; scale bar, 100 μm). At this developmental stage, all embryos are still alive (Spr embryos die at 70% DT, while BE and Fr die at about 90% DT) and do not differ consistently in morphology, although Spr embryos seem more compacted (multiple individuals were observed for each mutant). Arrows point to thoracic legs, and arrowheads indicate mouthparts.

Three spontaneous mutants with disturbed eyespot size and/or colour composition have been isolated from laboratory populations of *B. anynana*. Relative to 'wild-type' (WT) butterflies, Bigeye (BE) mutants have dramatically enlarged eyespots [21,41], Frodo (Fr) have eyespots with a broader outer golden ring, and Spread (Spr) have very large eyespots with gold scales almost completely replacing the black scales (Figure 1a).

The failure to establish pure-breeding stocks of these mutants suggested embryonic lethality, similar to that described for the eyespot mutant Goldeneye [18]. We compared proportions of mutant embryos and adults in experimental crosses to those expected if BE, Fr and Spr phenotypes were caused by embryonic recessive lethal alleles with a dominant effect on eyespot pattern. In this situation, crosses between individuals of the same mutant phenotype should result in one fourth of the offspring dying during embryogenesis, and two thirds of the adults having aberrant eyespots. On the other hand, crosses between mutant and WT butterflies should yield no embryonic lethality and one half of the adults with aberrant eyespots. The results of all crosses, including statistical analysis, are summarized in Table 1. As expected, crosses between mutant individuals of the same phenotype resulted in 1:2 segregation of WT and mutant adults (33.6 and 66.4%, respectively, in a total of 1720 adult progeny from 39 families), while crosses between each mutant and WT butterflies resulted in 1:1 segregation (47.4 and 52.6%, in 2113 adult offspring from 28 families). Consistent with BE, Fr and Spr phenotypes being due to recessive lethal alleles, approximately one fourth of the progeny of crosses between same-phenotype mutants died during embryogenesis (25.3% of 5526 progeny in 39 families), while no aberrant embryos were found in crosses to WT butterflies.

**Table 1:** Segregation of aberrant embryos and adults in experimental crosses

Cross	No. fam	No. progeny	Aberrant embryos			No. adult progeny with eyespot phenotypes					
			**% [SD]**	**χ**^**2 **^_**HOM **_**[P]**	**χ**^**2 **^_**GF **_**[P]**	**WT**	**BE**	**Fr**	**Spr**	**χ**^**2**^_**HOM **_**[P]**	**χ**^**2**^_**GF **_**[P]**

BE × BE	16	2954	24.0 [2.9]	12.89 [0.6107]	1.68 [0.1949]	250	521	0	0	3.70 [0.9986]	0.28 [0.5967]

Fr × Fr	10	1156	27.4 [3.1]	4.98 [0.8373]	3.61 [0.0574]	175	0	334	0	3.38 [0.9473]	0.25 [0.6171]

Spr × Spr	13	1416	26.4 [4.3]	8.97 [0.7065]	1.51 [0.2191]	0	153	0	287	4.60 [0.9700]	0.41 [0.5220]

BE^2 ^× BE^2^	8	1488	22.9 [2.2]	4.03 [0.7765]	3.44 [0.0636]	151	278	0	0	13.99 [0.0514]	0.67 [0.4131]

BE^3 ^× BE^3^	4	667	3.1 [2.3]	10.78 [0.0292]	-	0	393	0	0	-	-

BE × WT	14	2459	0	-	-	499	549	0	0	18.58 [0.1367]	2.39 [0.1221]

Fr × WT	5	659	0	-	-	227	0	249	0	0.69 [0.9526]	1.02 [0.3125]

Spr × WT	9	1272	0	-	-	275	314	0	0	4.68 [0.8613]	2.59 [0.1075]

BE^3 ^× WT	11	-	-	-	-	1486	0	0	0	-	-

BE × Fr	9	1671	24.4 [4.1]	10.24 [0.2486]	0.31 [0.5777]	185	185	164	0	-	-

BE × Spr	24	2974	23.6 [4.8]	27.26 [0.2451]	3.05 [0.0807]	408	480	0	412	-	-

BE^3 ^× BE	7	1092	1.0 [4.0]	90.98 [0.0000]	-	356	0	0	310	-	-

The ratios of aberrant embryos and adults were tested for variation among families within each cross with χ^2 ^test for homogeneity (χ^2 ^_HOM _with d.f. = N_fam _- 1). When ratios were not significantly different across families, the numbers of progeny were pooled and tested against the expected ratios with χ^2 ^goodness-of-fit test (χ^2 ^_GF _with d.f. = 1). Otherwise, the ratios were tested against the expected values in each family separately (d.f. = 1; see "Different BE phenotypes and variation in lethality of underlying alleles" section). Standard deviation (SD) and *P *values (P) are given in square brackets.

The aberrant embryos in the BE × BE, Fr × Fr, or Spr × Spr crosses showed severe and very similar morphological defects, suggesting that all three mutations are alleles of the same locus. This was confirmed by complementation tests: crosses between BE and either Fr or Spr mutants yielded embryonic lethality in approximately one fourth of the offspring (23.9% of 4645 progeny in 33 families), with identical morphological aberrations as those found in the offspring of crosses between mutants of the same phenotype. In contrast, no embryonic lethality or aberrant morphology was observed in 1756 progeny of 13 crosses between BE and Goldeneye individuals. This shows that these two mutations occurred in different genes, which is also consistent with the fact that the embryonic phenotype caused by the *Goldeneye *allele (i.e., disturbed blastokinesis [18]) is different from the embryonic effects produced by the alleles underlying the BE, Fr or Spr phenotypes (see "Analysis of aberrant embryos reveals defects in segment polarity" section).

### Different BE phenotypes and variation in lethality of underlying alleles

Dramatic effects on eyespot morphology in BE, Fr and Spr mutants seem to be caused by different dominant alleles at the same locus, each disturbing embryonic development in homozygotes. However, the inheritance mode of the Spr phenotype appears to be more complex. The effect of the underlying allele on eyespot *colour composition *but not *size *is recessive, since offspring from Spr × WT crosses either have 'wild-type' eyespots or large eyespots with 'normal' colour scheme (46.7 and 53.3%, respectively, in 1272 adult offspring from nine families; Table 1). The latter phenotype is indistinguishable from that of BE individuals and is hereafter referred to as BE^2^. A similar phenotype, hereafter called BE^3^, is found in the 'non-Spr' progeny from Spr × Spr crosses (34.8% of 440 adults in 13 families). Single-pair crosses were set up to determine whether enlarged eyespots in BE individuals and in the offspring of Spr × WT (BE^2 ^individuals) and Spr × Spr crosses (BE^3 ^individuals) were caused by the same or by different alleles (Table 1).

Similarly to BE × BE, crosses between two BE^2 ^individuals yielded embryonic lethality in approximately one fourth of the progeny (22.9% of 1488 progeny in eight families) and enlarged eyespots in two thirds of the adults (64.8% of 429 adult offspring). These results are consistent with BE and BE^2 ^phenotypes being due to the same embryonic lethal allele with a dominant effect on eyespot size. In contrast, only a small fraction of embryos from crosses between two BE^3 ^individuals died before hatching and showed typical morphological defects (Table 1). This ratio was significantly lower than the expected 25% (**χ^2 ^**test of homogeneity among four families = 10.78, *P *< 0.05; **χ^2 ^**goodness-of-fit for each family = 53.05, 22.00, 29.37, 42.29, all with *P* < 0.05) and varied between 0 and 5.3%, perhaps due to modifier loci or incomplete penetrance. No embryonic lethality was observed in five of the seven crosses between BE^3 ^and BE individuals. The fraction of aberrant embryos in the other two families (10.8 and 1.1%) was significantly lower than the 25% expected if the BE^3 ^and BE phenotypes were produced by the same lethal allele (**χ^2 ^**test for homogeneity among seven families = 90.98; **χ^2 ^**goodness-of-fit in two families = 20.79 and 7.55; *P *< 0.01). This suggests that the mutation underlying the BE^3 ^phenotype is a different allele. It has a mildly deleterious effect on embryogenesis (i.e., low incidence of embryonic mortality) and a recessive effect on eyespot size, since all offspring of the BE^3 ^× WT crosses (i.e., BE^3 ^heterozygotes) have 'wild-type' eyespots (Table 1).

**Figure 2:**
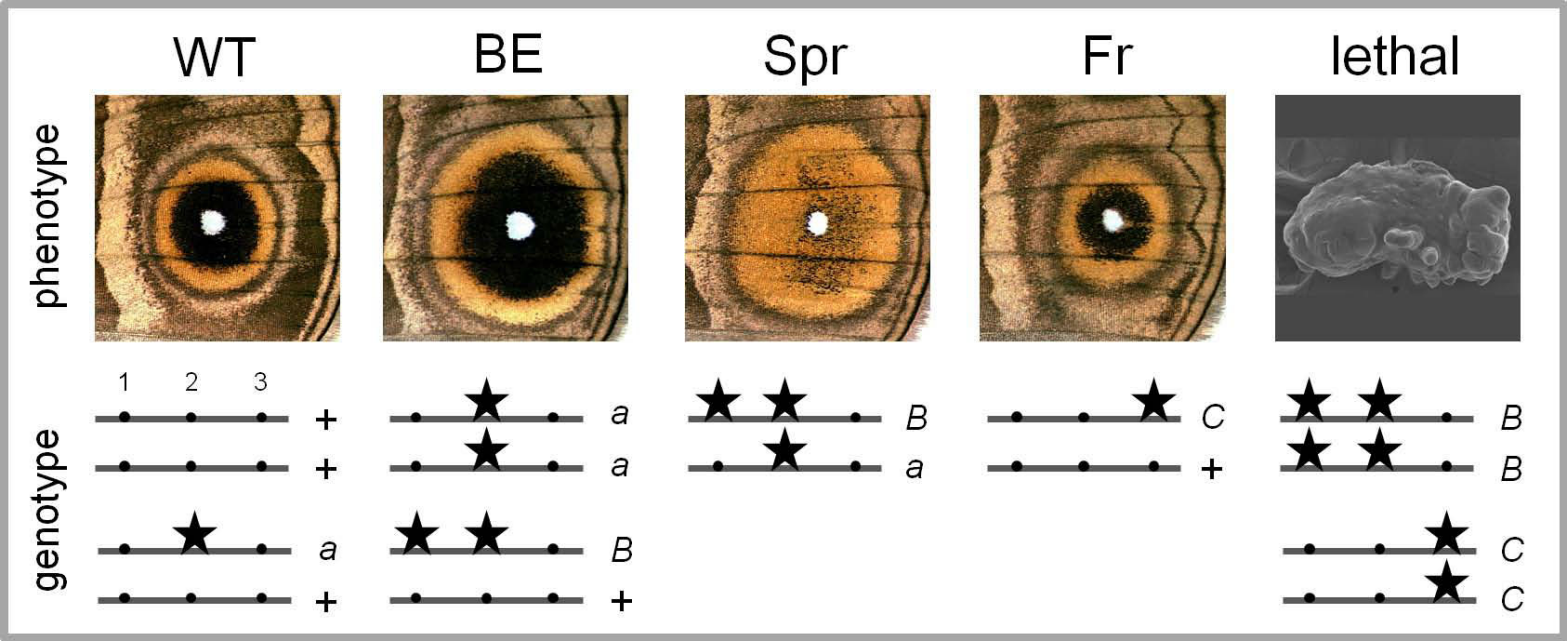
**Model for the embryonic and eyespot effects of the *BE*/*Fr*/*Spr *locus**. For each mutant phenotype, possible genotypes are shown with lines representing the locus, dots corresponding to different sites therein, mutations indicated with stars (the order and the distance between sites is arbitrary), and labels +, *a*, *B *and *C *representing the wild-type and three mutant alleles. Mutations at these three sites, isolated or in combination, define different alleles which can explain all our data. A single copy of the *BFS^a ^*allele has no obvious effect on eyespot morphology (the WT phenotype), but two copies produce enlarged eyespots which have 'normal' colour composition (the BE^3 ^phenotype; phenotypically indistinguishable from BE/BE^2^). Mutations at sites 1 and 2 together make up the *BFS^B ^*allele; it has a dominant effect on eyespot size (the BE/BE^2 ^phenotype), and, in combination with the *BFS^a ^*allele, affects colour composition (the Spr phenotype). This explains the recessive colour composition aspect of Spr inheritance and the presence of BE^3 ^individuals (*BFS^a ^*homozygotes) in crosses between two Spr individuals (Table 1), and is consistent with Spr having been isolated from the BE stock (see Materials and Methods). A mutation at a third site in this locus (corresponding to the *BFS^C ^*allele) affects eyespot ring boundaries (the Fr phenotype). The alleles *BFS^B ^*and *BFS^C ^*are embryonic recessive lethal and display a segment polarity phenotype.

On the basis of the segregation of eyespot and embryonic mutant phenotypes in the experimental crosses, we propose that the three mutations occurred in the *BE*/*Fr*/*Spr *(*BFS*) locus, and that two of them are embryonic recessive lethal. We suggest that different combinations of the wild-type (*BFS^+^*) and the three mutant alleles underlie the observed eyespot and embryonic phenotypes (Figure 2). The BE/BE^2 ^phenotype (each obtained in a different cross; see Table 1) is due to a single copy of the recessive lethal *BFS^B ^*allele, the identical BE^3 ^phenotype is caused by the recessive nonlethal *BFS^a ^*allele, and the Spr phenotype, by a combination of both. The Fr phenotype is produced by a single copy of another recessive lethal allele, *BFS^C^*. In this model, alleles *BFS^a ^*and *BFS^C ^*each carry a mutation at a single site, while the *BFS^B ^*allele carries two, one shared with *BFS^a^*. This is consistent with the fact that the Spr mutant (genotype *BFS^a^*/*BFS^B^*) was originally isolated from the BE stock (see Materials and Methods), and it explains the data from all our crosses, including the more complex inheritance we described for Spr, and the different "enlarged eyespots" phenotypes we characterized (see previous section). Specifically (see Table 1), (1) crosses between two Spr individuals segregate for Spr and BE^3 ^phenotypes (and not for Spr and WT, as would be the case if the Spr phenotype was due to a single, independent mutation at the *BFS *locus), and (2) the Spr phenotype is lost in progeny from Spr × WT crosses (showing that it is recessive), but recovered in progeny from BE^3 ^× BE crosses (showing that BE individuals carry the relevant mutation) (see Figure 2).

### Analysis of aberrant embryos reveals defects in segment polarity

Compared to WT embryos of the same age, homozygotes for each of the lethal alleles displayed severe and similar abnormalities. They were much shorter and thicker than WT embryos (Figure 1b). The typical 3 thoracic and 10 abdominal segments were all present, as was clear from the number of thoracic and abdominal appendages, but the segments were compressed and their borders poorly defined. Dorsal closure was not completed in approximately 30% of the embryos. The thoracic legs and the mouthparts (arrows and arrowheads in Figure 1b) were short; some embryos were missing one leg while one of the remaining legs was branched. We observed variation in timing of death among mutants. Typically, embryos from Spr × Spr crosses died before the stage when bristles appear (~70% of developmental time, DT [42]), and looked more compacted, while embryos from crosses between BE or Fr butterflies were fully sclerotized and had melanized head capsules, and died at approximately 90% DT. The severe shortening of segments and poorly defined segment boundaries observed in all mutant embryos suggest that the mutations affect the structure of each segment, rather than their establishment, possibly by interfering with the normal function of segment polarity genes.

**Figure 3:**
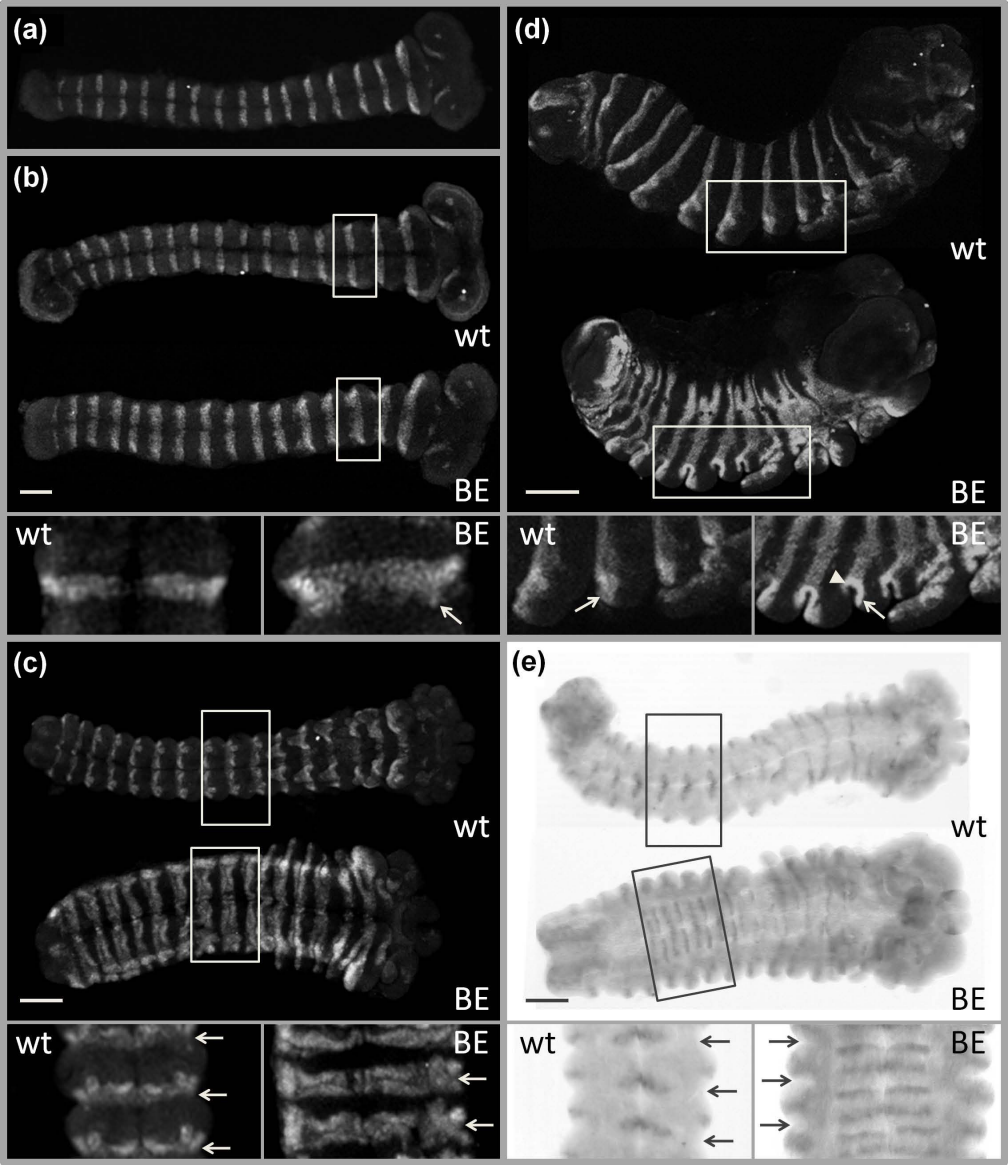
**Expression of segment polarity genes in embryos**. (a) Ventral view of an embryo from the BE stock (including both BE and WT embryos) at 12% DT; *en *is expressed in the posterior compartment of each segment, which shows that the establishment of its expression is not affected. (b) Ventral view of a WT and a BE embryo at 15% DT; En protein is also detected in some anterior cells of the segments (arrow) in BE embryos. (c) At 25% DT, BE embryos appear shorter than WT, with En present in the anterior and posterior cells of each segment (arrows indicate segment borders). (d) Lateral view of a WT and a BE embryo at 40% DT. Arrows point to posterior cells of the second abdominal segment, expressing *en*. In BE embryos, En is also detected in anterior cells of each segment (arrowhead). (e) *wg *expression in embryos at 25% DT (arrows indicate segment borders). In WT, *wg *mRNA is detected in a single stripe per segment, while an extra *wg *stripe is present in each segment in a BE embryo. Segment borders allow a clear assessment of the relative position of *en *and *wg *expression stripes. Anterior is to the right; scale bar, 100 μm. Mutant embryos from Spr or Fr stocks show identical patterns of *en *and *wg *expression.

Analysis of expression patterns of key segment polarity genes *engrailed *(*en*) and *wingless *(*wg*) revealed substantial differences between WT and mutant embryos. In early embryos (Figure 3a), *en *was expressed in the posterior compartment of each segment, as is typical for insects [43]. No aberrations in morphology or *en *expression were detected in 85 embryos dissected at 12-13% DT, revealing no differences among mutant and WT embryos. At 15% DT, the differences became obvious with the En protein detected not only in the posterior compartment, as in WT, but also in the anterior cells of each segment in mutant embryos (Figure 3b). Defects in segmentation became even more apparent at 25% DT, when mutant embryos appeared shorter due to compacted segments, and the En stripes were almost twice as broad as those of WT (Figure 3c). At 40% DT, mutant embryos looked almost spherical, with En clearly visible in a posterior and an anterior stripe within each segment, thoracic appendages, and abdominal prolegs (Figure 3d). Differences in *wg *expression were also very clear at 25-40% DT. In WT, *wg *mRNA was detected in each segment in stripes positioned just anterior to the En domain, while in mutant embryos two stripes of *wg *were observed in each segment, with the additional stripe positioned posterior to the expanded En domain (Figure 3e).

These results show that the lethal alleles at the *BFS *locus do not affect the specification of segment number or the establishment of *en *and *wg *expression domains in embryonic segments, but rather disturb their correct maintenance and generate a typical segment polarity phenotype, i.e., the 'replacement' of the anterior part of each segment by a mirror image duplication of the posterior part [44]. In *Drosophila melanogaster*, identical embryonic defects are caused by loss-of-function mutations, or experimental knockdown, of one of the negative regulators of Wg signalling; *zeste-white 3 *[45], *naked cuticle *[46], *axin *[47], *APC2 *[48], or *Bili *[49].

### Epidermal response properties are affected in the Spr mutant

The formation of eyespots in developing pupal wings involves the production and diffusion of a morphogen from the cells at the center (stage called 'focal signalling') and the response of the surrounding epidermal cells [31,32]. Different levels of the signal induce expression of different regulatory genes, e.g., *Distall-less *(*Dll*) and *spalt *(*sal*) in the inner disc, and *en *in the outer ring [17], which then directly or indirectly control biosynthesis of different pigments. Changes in the focal signal (e.g., morphogen concentration, stability or diffusion coefficient) have been shown to explain most of the variation in eyespot size between artificial selection lines [50], while the sensitivity levels of the epidermal cells to that signal determine eyespot colour composition [20,51] and, to a lesser extent, eyespot size [21,50]. Genetic correlations between eyespot size and colour composition are typically low [50-52], suggesting that different sets of genes determine these two aspects of eyespot morphology, and their underlying components of pattern induction.

**Figure 4:**
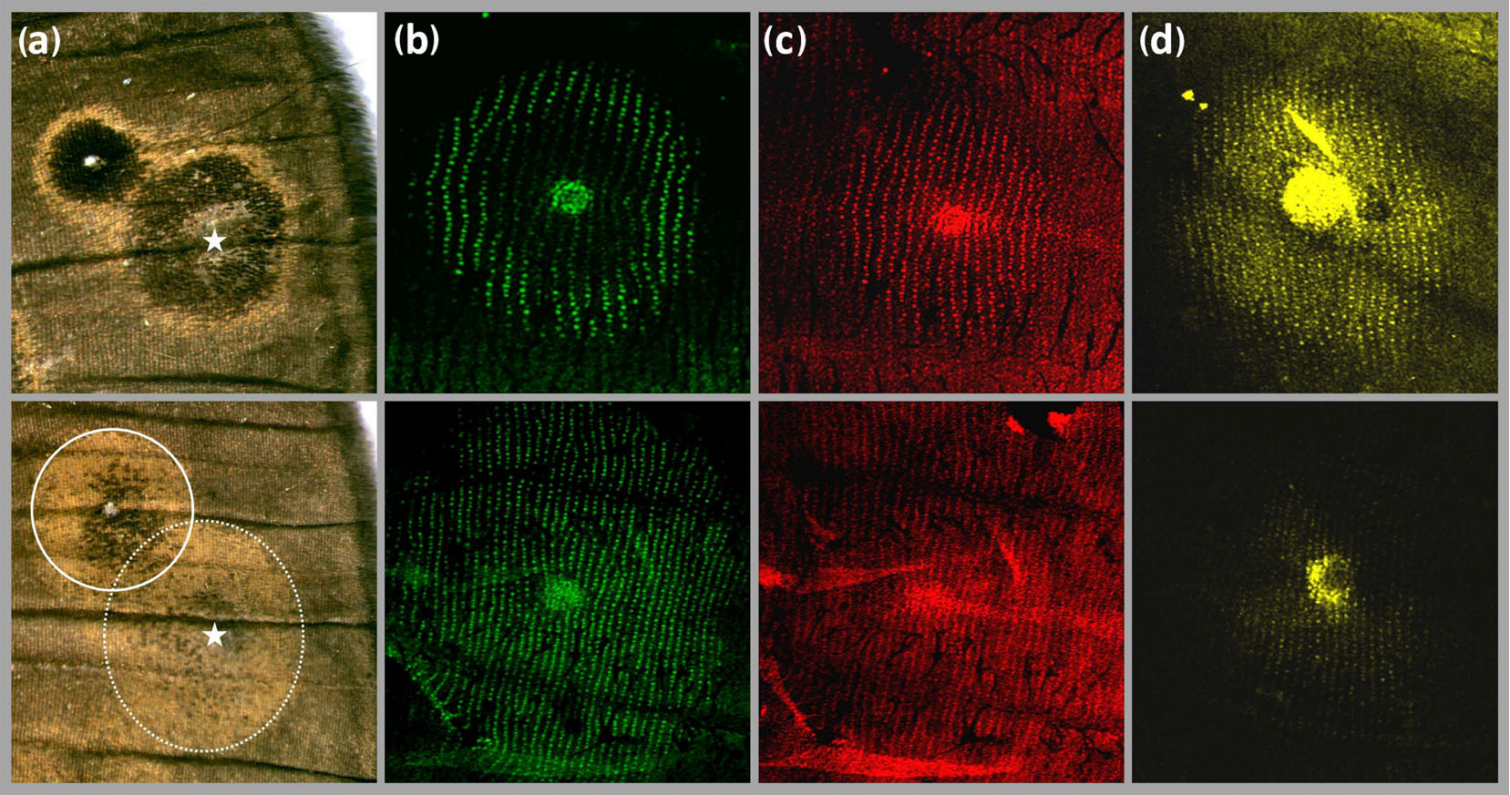
**Ectopic eyespots and expression of eyespot patterning genes**. Eyespots of WT (top) and Spr individuals (bottom) differ in colour scheme in adults, and in gene expression patterns in developing pupal wings. (a) Anterior distal part of dorsal adult forewings showing native and damage-induced eyespots formed around wound sites (stars). The colour composition of the ectopic eyespots resembles that of the native eyespots and reveals differences in the response properties of wing epidermal tissue between WT and Spr butterflies (solid and dashed rings outline the Spr native and the ectopic eyespots, respectively). These differences are reflected in the expression patterns of *en *(b), *Dll *(c) and *sal *(d) in 16- to 18-hr old pupal wings. Spr mutants were chosen for these experiments because they exhibit the most severe, and thus most noticeable, effects on eyespot phenotype, including damage-induced ectopic eyespots.

Damage applied to the developing wing in early pupa typically induces the formation of ectopic patterns [53], and can be used to probe variation in epidermal response sensitivities (see [20]). Damage-induced ectopic eyespots were produced in more than 60% of cauterized WT and Spr pupae (32 of 50 for WT, and 56 of 70 for Spr), and typically resembled the native eyespots on the same wing surface (Figure 4a). Damage-induced eyespots consisted of a black inner disc and an outer golden ring in WT, but were almost entirely made up of gold scales in Spr. These results show that the ability of the entire wing epidermis to respond to eyespot-inducing signals, such as damage, is altered in Spr mutants. We also show that the expression patterns of 'response' eyespot ring genes were altered in Spr wings: *en *was detected in almost all cells of the eyespot field (Figure 4b), while the numbers of scale-building cells expressing *Dll *and *sal *were strongly reduced (Figures 4c and 4d). Taken together, our results are consistent with an effect of the target gene downstream of the eyespot-inducing focal signalling, but upstream of the response patterning genes.

### The Wnt/Wg signalling pathway and butterfly eyespot formation

Our study of embryonic defects and of *en*/*wg *expression in the target *B. anynana *mutants suggests that the *BFS *locus is a negative regulator of the Wnt/Wg signalling pathway. Analysis of eyespot formation in Spr wings further suggests that the product of this gene acts upstream of eyespot ring patterning transcription factors during the 'response to focal signal' stage. The Wg morphogen, implicated in the evolution of wing pigmentation spots in *Drosophila *[54], has been proposed as a candidate focal signal in butterflies [35]. It is produced by eyespot centres in early pupal wings of *B. anynana *[35], and is known to upregulate *en *and *Dll *in insect embryos and imaginal wing discs, respectively [45,55]. In butterfly wings, Wg could act as the inducer of the circular *Dll *and *en *domains corresponding to eyespot rings in adults [17]. Mutations in a negative regulator of the Wnt/Wg pathway, as could be the *BFS *gene, would affect the response of epidermal cells to this signal, altering *en *and *Dll *expression and therefore the distribution of black and gold scales. Molecular identification and functional characterization of the *BFS *locus, including the analysis of expression of key components of Wg signalling in both normal and altered eyespot development, will be necessary to assess its role in the regulation of this signalling pathway.

In a previous study, we mapped the *BFS *locus to approximately a 1.3 Mb interval on *B. anynana *chromosome 17, which has a high level of synteny with its ortholog in *Bombyx mori *[37], the model lepidopteran with the fully sequenced and annotated genome. Curiously, the orthologous chromosome in the butterfly *Heliconius melpomene *carries colour pattern loci implicated in intra- and interspecific divergence [56]. Inspection of gene content in the genomic regions of *B. mori *(nscaf2829 [57]) and *H. melpomene *[56], orthologous to the *BFS *interval, revealed that they do not contain any of the candidate genes generated via the comparison of embryonic phenotype to *Drosophila *mutants (namely, *Axin*, *zeste-white 3*, *APC2*, *naked cuticle*, and *Bili*), or any other gene with a known role in the regulation of Wg signalling. The high levels of synteny between these lepidopterans [37] suggest that the implicated interval in *B. anynana*, even though its exact genetic content is still unknown, probably also does not contain any of the described negative regulators of the Wnt/Wg pathway. This pathway is crucial for embryonic development and numerous other processes, and is remarkably conserved and extensively studied (reviewed in [58-60]). Nevertheless, sophisticated new genetic and genomic approaches continue to identify new Wnt/Wg regulators [61,62], some of which are taxon-restricted (e.g., *WTX *[63]). The *BFS *locus could be another, yet unknown, negative regulator of this pathway, potentially specific to this lepidopteran lineage.

## Conclusions

Analysis of mutant alleles of large effect is a powerful approach for exploring the developmental basis of novel traits, and especially valuable for studies in nonmodel organisms with limited genetic resources. This work describes three pleiotropic mutations in a single locus, presumably a negative regulator of Wg signalling. The product of this gene is involved in the formation of eyespots, a butterfly-specific novelty, and a relatively conserved process of embryonic development [64-66]. Different alleles at this locus produce similar effects on embryonic segment polarity, but disturb different aspects of eyespot morphology. This locus maps to a genomic interval whose orthologous regions in two other, syntenic leidopterans do not contain any annotated genes with a known role in the regulation of the Wnt/Wg pathway. This suggests that the *B. anynana BFS *locus might be a yet undescribed negative regulator of Wg signalling.

**Figure 5:**
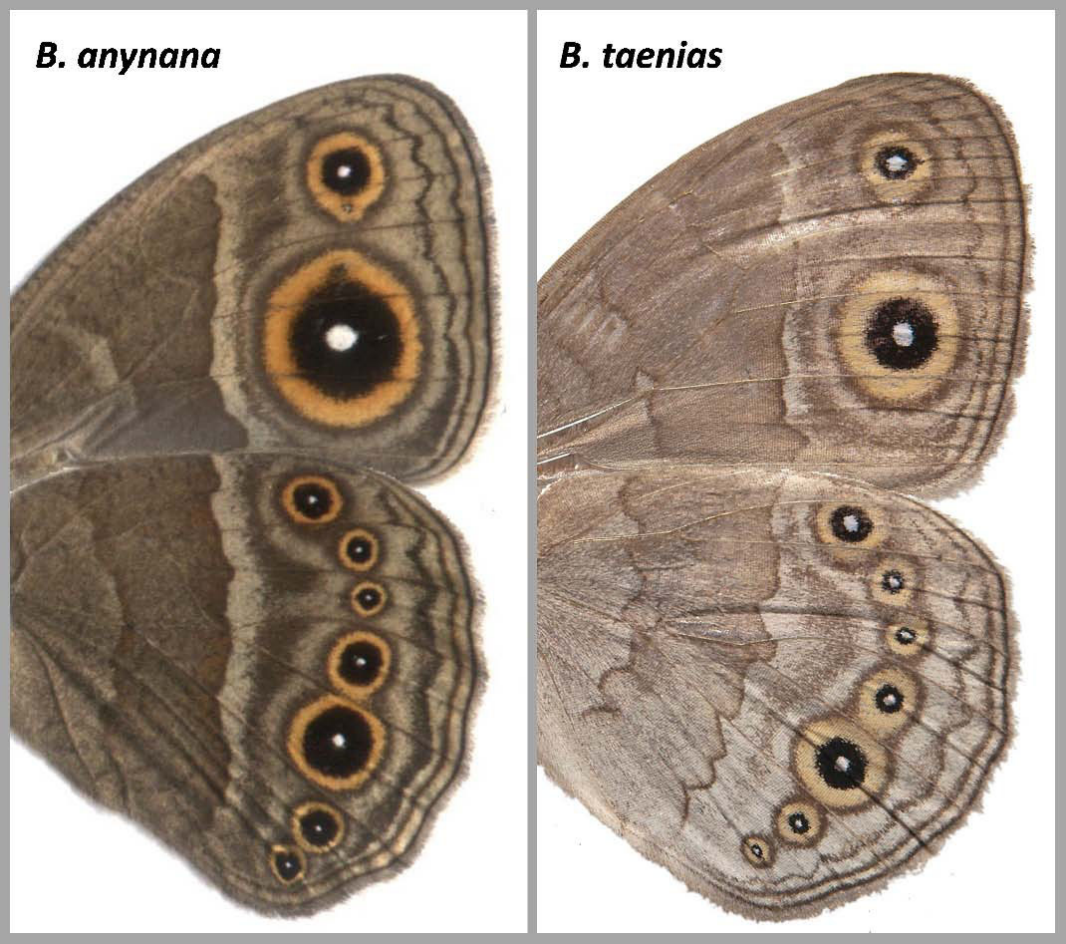
**Variation in eyespot size and colour composition in *Bicyclus***. The different phenotypes produced by allelic variation in our study locus resemble intra- and interspecific variation in the genus (compare to Figure 1a). The wing pattern of *B. anynana *lines artificially selected for large eyespots [69] resembles that of BE individuals. The *B. taenias *eyespots with broad golden rings resemble those of Fr butterflies. Further work is necessary to determine whether the same alleles or even loci underlie these different phenotypes.

Studies of lab mutants represent a valuable approach for understanding the developmental genetics of morphological traits, and can provide candidate genes for intra- and interspecific variation in natural populations [67,68]. Although it is unlikely that recessive lethal alleles at the *BFS *locus will contribute to naturally occurring variation in eyespot morphology, other alleles (e.g., the recessive mildly deleterious *BFS^a ^*allele) might. A striking feature of this locus is the diversity of effects the different alleles have on eyespot phenotype (i.e., on size and colour composition), either independently or together. Some of these resemble eyespot phenotypes obtained in artificial selection lines of *B. anynana *[20,69], and even those found in other species of the *Bicyclus *genus (examples in Figure 5). This raises the possibility that the locus we characterize here might be an exciting candidate gene for the diversification of this evolutionary novelty. Interestingly, our *B. anynana *eyespot locus is located in a genomic region [37] orthologous to that containing colour pattern loci in multiple other species and thought to be 'hotspots' for the evolutionary diversification of wing patterns [70].

Future work will shed light onto the exact nature and evolutionary significance of this interesting locus. Fine mapping can reveal whether it encodes a conserved or a derived regulator of Wg signalling and to what extent it is related to colour pattern loci in other butterflies. Analysis of its contribution to intra- and interspecific variation in eyespot morphology can address its role in the evolutionary diversification of butterfly wing patterns.

## Methods

### Butterfly stocks and crosses

All butterfly stocks were at reared 27°C as in [36]. The BE and Fr stocks were each set up from a single individual isolated from different laboratory populations in 1994 and 2007, respectively. The Spr stock was founded from a single individual isolated from the BE stock in 2003. All mutant stocks have been maintained with selection in favour of the mutant phenotype and, when necessary to avoid inbreeding depression, were outcrossed to the laboratory outbred WT stock.

Different crosses were set up to determine the mode of inheritance of the mutant alleles (Table 1). To test whether the mutations behave as embryonic recessive lethal alleles, butterflies from each mutant stock were crossed to individuals of the same phenotype and to WT stock butterflies. Crosses between butterflies of different mutant phenotypes were performed to determine whether the mutant alleles occur in the same locus (complementation tests). Eggs were collected from individual mating pairs, hatched larvae were reared through to adulthood, and the eclosed butterflies were frozen and scored for eyespot phenotype. Unhatched eggs were counted, dechorionated in 50% bleach solution for 1 min, rinsed with water, and fixed in 4% formaldehyde solution in 1 × phosphate-buffered saline (PBS). Embryos were dissected under a light microscope. Unfertilized eggs, identified as those lacking large blastoderm cells, were excluded from the analysis.

### Statistical analysis

χ^2 ^tests were used to compare observed proportions of mutant embryos and adults to those expected if the alleles underlying the BE, Fr and Spr phenotypes were embryonic recessive lethal with dominant effect on eyespot pattern. First, we tested whether the ratios varied among families using the χ^2 ^test of homogeneity. If ratios were not significantly different, the numbers of progeny were pooled over all families belonging to the same cross type and mutant phenotype; these overall frequencies were then tested against the expected ratios using the χ^2 ^goodness-of-fit test. If the χ^2 ^homogeneity test revealed significant variation among families, the frequencies of aberrant embryos and mutant adults were tested against the expected values separately in each of the families (the case for only BE^3^).

### Embryo morphology and gene expression analysis

Eggs were collected at different times after laying, dechorionated in 50% bleach solution for 1 min, rinsed with water, fixed in 10% formaldehyde in PBS/50 mM EGTA for 30 min, dehydrated in methanol in four steps and stored at -20°C until use. For scanning electron microscopy studies, 10-20 fixed embryos of each mutant phenotype were rehydrated in PBS in four steps and mounted on specimen holders, air-dried, coated with gold and viewed in a JEOL JSM 6400 scanning electron microscope.

For the analysis of gene expression patterns, 50-100 embryos were examined for each mutant line per developmental stage, defined according to the system developed for *M. sexta *[42]. Fixed embryos were rehydrated in PBS, blocked in PBT (5 mg/ml bovine serum albumin in PBS) for 2 hr, incubated with primary antibody overnight at 4°C (anti-Engrailed 4F11 [43]), washed 10 times in PBS with 0.1% Triton X-100 and incubated for 2 hr at 4°C in a 1:200 dilution of the secondary antibody (Alexa Fluor 488; Molecular Probes). After 10 washes in PBS, embryos were incubated in 100% glycerol for 1 hr and mounted on glass slides. Images were collected on a Bio-Rad MRC 1024 ES laser scanning confocal microscope.

A 315-bp fragment of *B. anynana wingless *gene (AY218276) was amplified from embryonic cDNA with primers 5'-GTCATGATGCCCAATACCG and 5'-GCAGTTGCATCGTTCCACTA and cloned into pCRII-TOPO dual-promoter vector using the TOPO TA cloning kit (Invitrogen). Plasmids were isolated with QIAprep Spin Miniprep Kit (Qiagen) and used as template for PCR reactions with vector primers M13F and M13R. The amplified products were cleaned with Wizard SV Gel and PCR Clean-Up System (Promega) and used for SP6 or T7 transcription. Sense and antisense digoxygenin-labeled riboprobes were synthesized using SP6 and T7 RNA polymerases and DIG RNA labeling mix (Roche Applied Science). The probes were run on an agarose gel and measured with NanoDrop spectrophotometer (Thermo Scientific) to verify their quality and concentration. *In situ *hybridization with sense and antisense probes was performed at 55°C for 48 hr following the protocol described previously [36]. The probes were detected with NBT/BCIP (Roche Applied Science). Stained embryos were mounted on slides and photographed with a Leica DC 200 digital camera attached to a Leica MZ 125 microscope.

### Induction of ectopic eyespots

Dorsal surface of left pupal forewings was damaged at 12-18 h (± 30 min) after pupation, when ectopic eyespots are produced most often [53]. Wings were pierced with a finely sharpened tungsten needle (cat. no. 501317; World Precision Instruments) at a site next to the small anterior eyespot, approximately halfway between the wing margin and the normal location of the eyespots. Operated pupae were returned to 27°C; adults were frozen soon after emergence, and their wings were photographed with a Leica DC 200 digital camera attached to a Leica MZ 125 microscope.

### Immunohistochemistry of pupal wings

Pupal wings of WT and Spr individuals were stained at 16-18 h after pupation according to the protocol described previously [17] with antibodies against Engrailed (4F11 [43], 1:50 dilution), Distal-less ([71], 1:200 dilution) and Spalt ([72], 1:500 dilution). Alexa Fluor 488 and Texas Red (Molecular Probes) were used as secondary antibodies in 1:200 dilutions.

## Authors' contributions

SVS and PB conceived the project, designed all experiments, and wrote the manuscript. SVS performed the experiments, and collected and analyzed all data. PB participated in data analysis and interpretation. PMB found the mutants and contributed to discussions on project design and data interpretation. All authors read and approved the final manuscript.
